# Paneth-like cells produced from OLFM4^+^ stem cells support OLFM4^+^ stem cell growth in advanced colorectal cancer

**DOI:** 10.1038/s42003-023-05504-8

**Published:** 2024-01-05

**Authors:** Mizuho Sakahara, Takuya Okamoto, Upasna Srivastava, Yasuko Natsume, Hitomi Yamanaka, Yutaka Suzuki, Kazutaka Obama, Satoshi Nagayama, Ryoji Yao

**Affiliations:** 1 Department of Cell Biology, Cancer Institute, Japanese Foundation for Cancer Research, Tokyo, Japan; 2 Department of Surgery, Graduate School of Medicine, Kyoto University, Kyoto, Japan; 3 Department of Computational Biology and Medical Sciences, Graduate School of Frontier Sciences, The University of Tokyo, Kashiwa, Chiba Japan; 4 Department of Surgery, Uji-Tokushukai Medical Center, Kyoto, Japan; 5 Present Address: Child Study Center, Yale School of Medicine, New Haven, CT USA

**Keywords:** Cancer stem cells, Cancer stem cells

## Abstract

Tumor tissues consist of heterogeneous cells that originate from stem cells; however, their cell fate determination program remains incompletely understood. Using patient-derived organoids established from patients with advanced colorectal cancer (CRC), we evaluated the potential of olfactomedin 4 (OLFM4)^+^ stem cells to produce a bifurcated lineage of progenies with absorptive and secretory properties. In the early phases of organoid reconstruction, OLFM4^+^ cells preferentially gave rise to secretory cells. Additionally, we found that Paneth-like cells, which do not exist in the normal colon, were induced in response to Notch signaling inhibition. Video recordings of single OLFM4^+^ cells revealed that organoids containing Paneth-like cells were effectively propagated and that their selective ablation led to organoid collapse. In tumor tissues, Paneth-like cells were identified only in the region where tumor cells lost cell adhesion. These findings indicate that Paneth-like cells are directly produced by OLFM4^+^ stem cells and that their interaction contributes to tumor formation by providing niche factors. This study reveals the importance of the cell fate specification program for building a complete tumor cellular ecosystem, which might be targeted with novel therapeutics.

## Abstract (web summary)

An analysis of the reconstitution process of orgenoids revelaed that OLFM4+ cells in colorectal cancer directrly generate Paneth-like cells, which supported OLFM4+ cells growth by providing niche.

## Introduction

Tumor tissues consist of heterogeneous cells whose organization plays a major role in tumor development^1–3^. The multilineage differentiation processes of the original normal tissue are thought to contribute to the cellular heterogeneity of the tumor tissue. In the normal intestine, enterocytes actively participate in nutrient absorption, whereas secretory cells control the conditions in the intestinal environment by releasing mucus and hormones. Stem cells present at the bottom of intestinal crypts give rise to highly proliferating transit amplifying (TA) cells, which in turn transform into absorptive cells and four principal secretory cell lineages: Paneth, goblet, enteroendocrine, and tuft cells^4,5^. Lateral inhibition of Notch signaling, which activates Hes family bHLH transcription factor 1 (HES1) and promotes the induction of absorptive progenitors, has been implicated in cell fate determination of absorptive and secretory lineages^6^. Conversely, cells with low Notch signaling express atonal BHLH transcription factor 1 (ATOH1), which drives their differentiation into secretory lineage progenitors^4^ and regulates downstream transcription factors to specify the secretory cell lineage^5^. Although comprehensive studies have been conducted on intestinal tissue in the mouse intestine, the cell fate determination program in human colorectal tissue has not been fully elucidated because of the lack of appropriate research platforms.

In addition to cellular differentiation, cel–cell interactions also facilitate tissue homeostasis by controlling cellular heterogeneity. This is best exemplified in the case of Paneth cells in the mouse small intestine, which reside at the bottom of crypts and support Lgr5^+^ stem cells^7,8^. In the colon, a subset of secretory lineage cells has been shown to support Lgr5^+^ stem cells, including c-Kit ^+^ colonic crypt base secretory cells^9^ and Reg4^+^ deep crypt secretory cells^10^. However, it remains unclear whether equivalent cells that support cancer stem cells exist in human colorectal cancer (CRC).

We have previously shown that patient-derived organoids (PDOs) established from patients with advanced CRC are composed of stem cell-like, TA cell-like, and differentiated cell-like clusters, which are further divided into subclusters based on their proliferation activity^11^. Olfactomedin 4 (OLFM4) was identified as the gene most strongly associated with the stem cell-like cluster. OLFM4^+^ cells exhibit self-renewal and multi-differentiation capabilities, highlighting their role as stem-like cells in advanced CRC. However, the types of differentiated cells produced from OLFM4^+^ cells and whether any progeny support OLFM4^+^ cells by providing a stem-cell niche remain unclear. In this study, we addressed these issues by examining the process of organoid reconstruction from single OLFM4^+^ cells.

## Results

### Production of OLFM4^−^ cells in the early phase of organoid reconstruction from single OLFM4^+^ cells

**Fig. 1:**
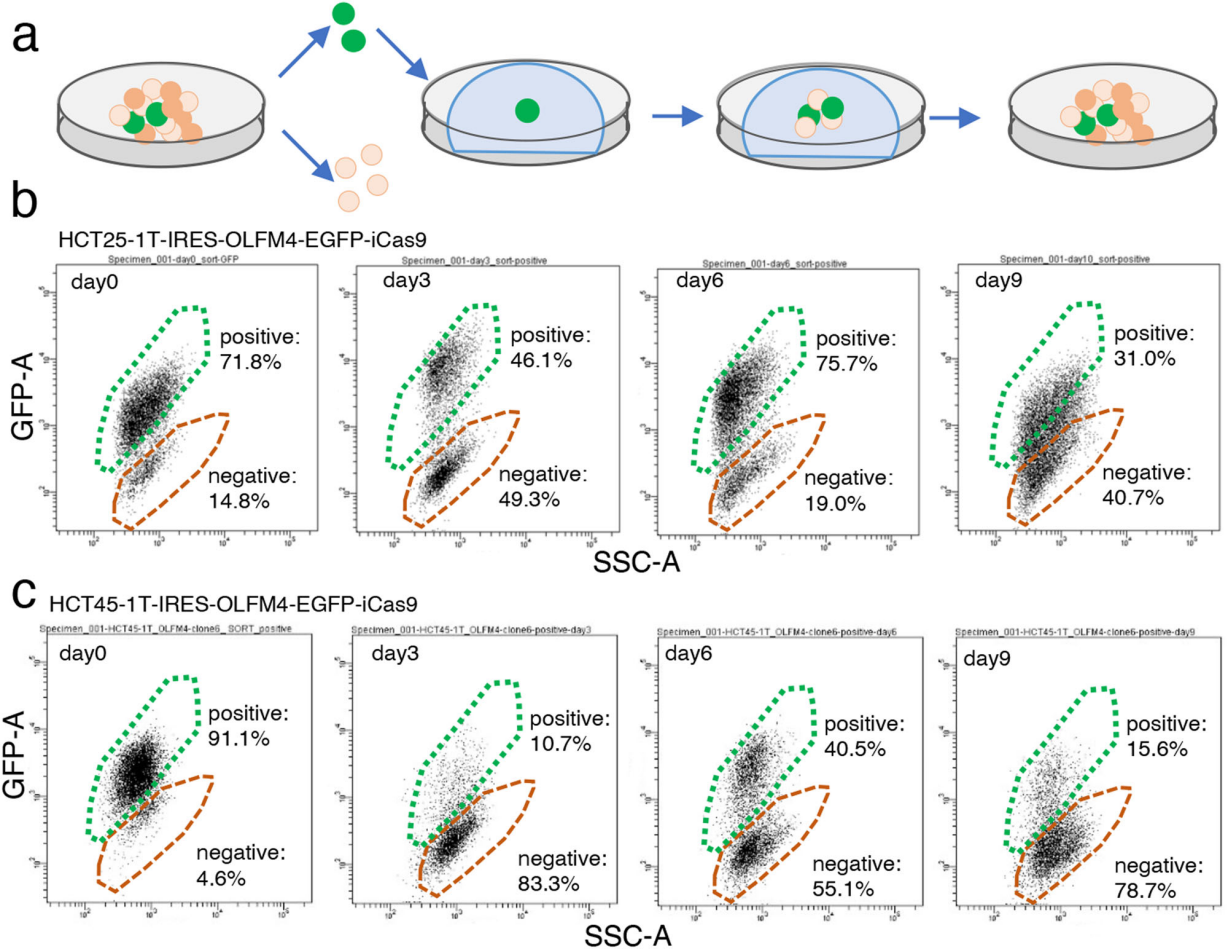
Reconstitution of organoids from single olfactomedin 4 (OLFM4)^+^ cells. **a** Schematic representation of the experimental design. Patient-derived organoids (PDOs) established from a patient with advanced colorectal cancer (CRC) containing the internal ribosome entry site-enhanced green fluorescence protein (IRES-EGFP)-P2A-inducible Caspase 9 (iCas9) cassette in the 3’-untranslated region (UTR) of the OLFM4 gene were dissociated into single cells. EGFP^+^ cells were sorted, cultured, and collected at 3, 6 and 9 days. Fluorescence-activated cell sorting (FACS) analysis of organoid reconstruction. Two PDOs established from different patients with CRC, HCT25 (**b**) and HCT45 (**c**), were analyzed. Representative figures of three independent experiments are shown.

To understand the cellular heterogeneity of CRC, we established PDOs from the tumor tissue developed in the ascending colon of a chemotherapy naïve patient with stage IV CRC (HCT25-1T). The organoid harbored APC (R216*), TP53 (IT123T), KRAS (G13D) and MSH6 (R1035*) mutations^11^. We partitioned this PDO into five clusters, including stem-like, TA-like, and differentiated cell-like clusters, using single cell RNA sequencing (scRNA-seq) analysis as described previously^11^. OLFM4 was identified as a stem cell marker, and OLFM4^+^ cells exhibited self-replication and multi-differentiation potentials. We established PDOs harboring the internal ribosome entry site-enhanced green fluorescence protein (IRES-EGFP)-P2A-inducible Caspase 9 (iCas9) cassette (EiC) in the 3’-untranslated region (UTR) of the OLFM4 locus^11,12^. OLFM4^+^ cells were identified by their EGFP expression, and BB homodimerizer, a cell-permeable ligand that dimerizes iCas9, selectively eliminates OLFM4^+^ cells^13^. The cell clusters were dissociated into single cells and sorted using fluorescence-activated cell sorting (FACS), and EGFP^+^ cells were embedded in Matrigel (Fig. 1a). On days 3, 6, and 9 after plating, the cells were collected and EGFP expression was analyzed. On day 3, the percentage of EGFP^+^ cells was reduced from 71.8% to 46.1%, which recovered to 75.7% on day 6 and decreased further to 31.0% on day 9 (Fig. 1b). Similar results were obtained with PDOs established from a different chemotherapy naïve patient with CRC, HCT45-1T (Fig. 1c), which harbored APC (R481*) and TP53 (H140HI) mutations^11^. It is less likely that the increased number of EGFP^-^ cells in the early phase of the reconstitution process was caused by self-replication of EGFP^-^ cells, because their organoid formation efficiency was approximately six times lower than that of EGFP^+^ cells^11^. These observations suggest that a subset of OLFM4^−^ cells were produced in the early phase of organoid construction.

### Cellular heterogeneity of PDOs reflects the composition of normal intestinal tissue

**Fig. 2:**
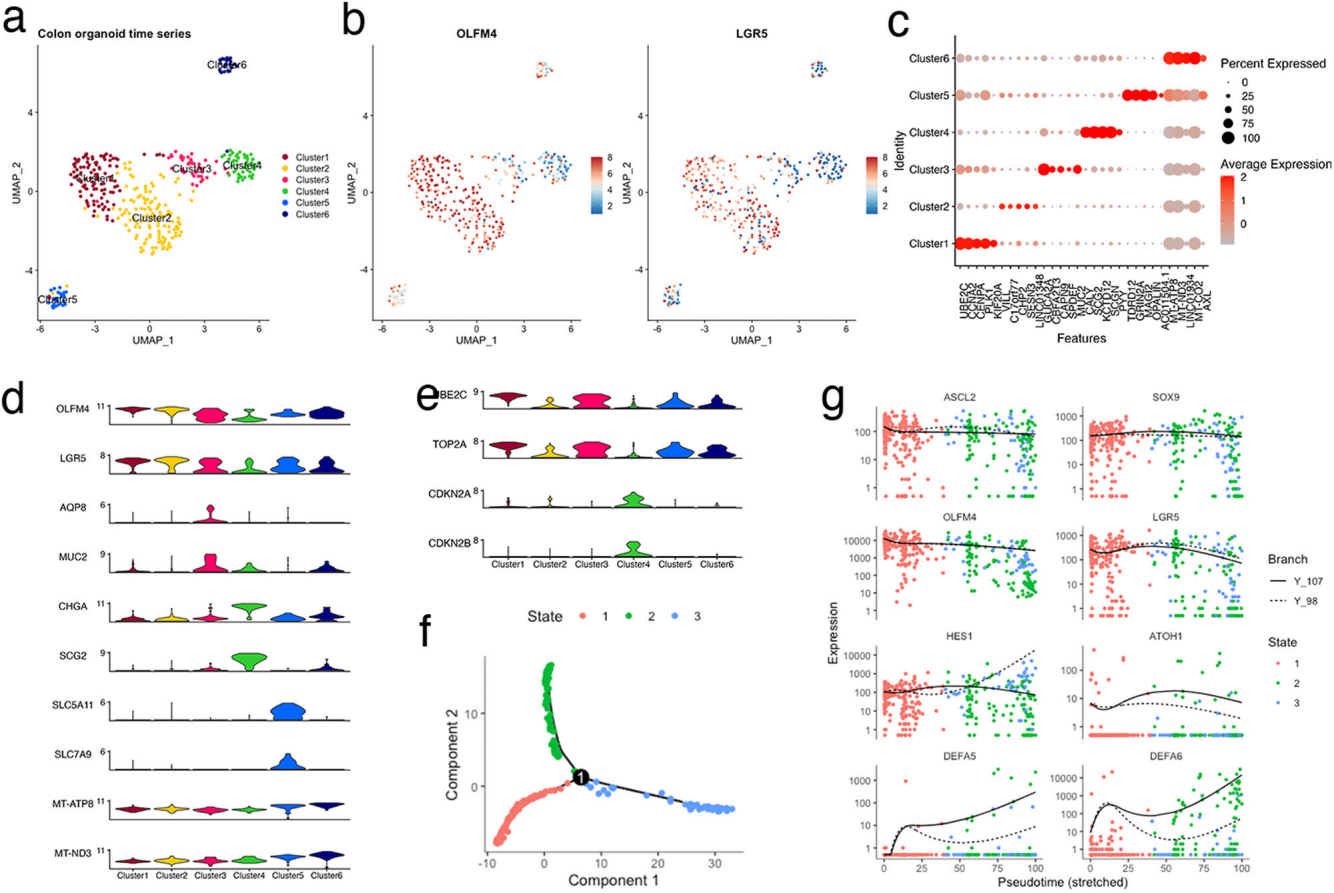
Cellular heterogeneity of patient-derived organoids (PDOs) from a patient with advanced colorectal cancer (CRC). **a** Cell-type clusters. Fluorescence-activated cell sorting (FACS)-sorted EGFP^+^ cells of HCT25-1T harboring the internal ribosome entry site-enhanced green fluorescence protein (IRES-EGFP)-P2A-inducible Caspase 9 (iCas9) cassette in the 3’-untranslated region (UTR) of the olfactomedin 4 (OLFM4) locus were plated, and organoids were collected at indicated days. Media were exchanged on days 0, 3, and 6. Single-cell RNA sequencing (scRNA-seq) data on days 0, 3, 6, and 9 were combined and cell clusters were visualized using uniform manifold approximation and projection (UMAP). Colors indicate the six clusters. **b** Expression level of OLFM4 and LGR5 plotted on UMAP. The color key from blue to tangerine indicates the relative gene expression levels from low to high. **c** Average expression level of the top 5 differentially expressed genes. The color of each dot represents the average expression level, and the size of each dot represents the ratio of positive cells for each gene. **d**, **e** Violin plot showing the expression level of canonical marker genes of intestinal cells (**d**) and cycling and senescent cells (**e**). **f** Pseudotime trajectory analysis indicating the state of all cells from the six clusters. Predicted stem-like cells are in red, absorptive-like cells are in blue, and secretory lineage-like cells are in red. **g** Cells in each cluster plotted on pseudotime trajectory analysis.

To analyze the cell types or states during organoid reconstitution, cells were collected on days 0, 3, 6, and 9 after plating and profiled using scRNA-seq analysis. The cells were partitioned into six groups via unbiased clustering of combined timepoint data, and visualized using uniform manifold approximation and projection (UMAP) (Fig. 2a). The majority of OLFM4^+^ cells were associated across two clusters, clusters 1 and 2. The cells in these clusters also expressed leucine-rich repeat-containing G-protein coupled receptor 5 (LGR5), which confirmed the presence of stem-like cell clusters (Fig. 2b). Investigation of differentially expressed genes (DEGs) for each cluster identified UBE2C and CCNA2 as marker genes for cluster 1 (Fig. 2c). Analysis of the Molecular Signatures Database revealed that gene sets related to the cell cycle and mitosis were positively and negatively enriched in clusters 1 and 2, respectively (Supplementary Fig. 1). These expression profiles indicated that OLFM4^+^ cells were classified into mitotically active and inactive states.

To further define clusters, the expression of canonical marker genes in adult intestines and cell cycle was examined (Fig. 2d, e)^4,14^. The goblet cell makers, AQP8 and MUC2, and enteroendocrine cell markers, CHGA and SCG2, were specifically upregulated in clusters 3 and 4, respectively. Absorptive enterocytes were partitioned into cluster 5 based on the expression of SLC5A11 and SLC7A. Cells in cluster 6 exclusively expressed higher levels of mitochondrial genes, suggesting that they were dying or of low quality. Overall, these analyses demonstrated that OLFM4^+^ cells produce principal absorptive and secretory lineage cells that constitute the normal intestinal tissue.

### Cellular lineage of organoids established from single OLFM4^+^ cells recapitulate the cell specification program of normal intestinal tissues

Trajectory inference analysis revealed three cellular states with bifurcating lineages (Fig. 2f). Cells in state 1 mainly contained clusters 1 and 2, suggesting a stem-like cell state (Supplementary Fig. 2). Cells in clusters 3 and 4 were assigned to state 2, whereas those in cluster 5 were assigned to state 3. These data suggest that the trajectory arose from stem-like cells (state 1), which separated into secretory (state 2) and absorptive enterocyte lineages (state 3). This cellular lineage faithfully recapitulates the cellular differentiation hierarchy of the normal human colon^15^.

Intestinal cell specification is controlled by the hierarchical expression of lineage-specific transcription factors^5^. ASCL2 and SOX9 are direct target genes of WNT signaling and function to maintain stem cells by repressing differentiation^16^. In CRC-derived PDOs, these transcription factors and the stem cell markers OLFM4 and LGR5 remained relatively constant among states (Fig. 2g), representing the constitutive activation of WNT signaling by bi-allelic APC truncation mutations of this organoid^11^.

HES1 and ATOH1 were upregulated in states 3 and 2, respectively (Fig. 2g), suggesting that the bilateral inhibition of Notch signaling controls cellular specification in advanced CRC. Secretory cell lineage markers and the transcription factors involved in secretory cell lineage specification were preferentially expressed in state 2 (Supplementary Fig. 3a)^5,17,18^. The kinetics of the endocrine cells varied (Supplementary Fig. 3b), reflecting the multiple subtypes of this lineage^18^. Tuft cell marker DCLK1 and its transcription factor, POU2F3, were preferentially expressed in state 3 cells (Supplementary Fig. 3c). These observations are consistent with a recent study reporting that tuft cells are specified outside the ATOH1-dependent secretory lineage^19^. Overall, the data from the trajectory inference analysis of reconstituting organoids provide evidence that advanced CRC-derived PDOs contain multiple cell types that resemble those observed in the normal intestine, and that key transcription factors that control cellular specification are largely conserved.

### Induction of Paneth-like cells during organoid reconstitution

**Fig. 3:**
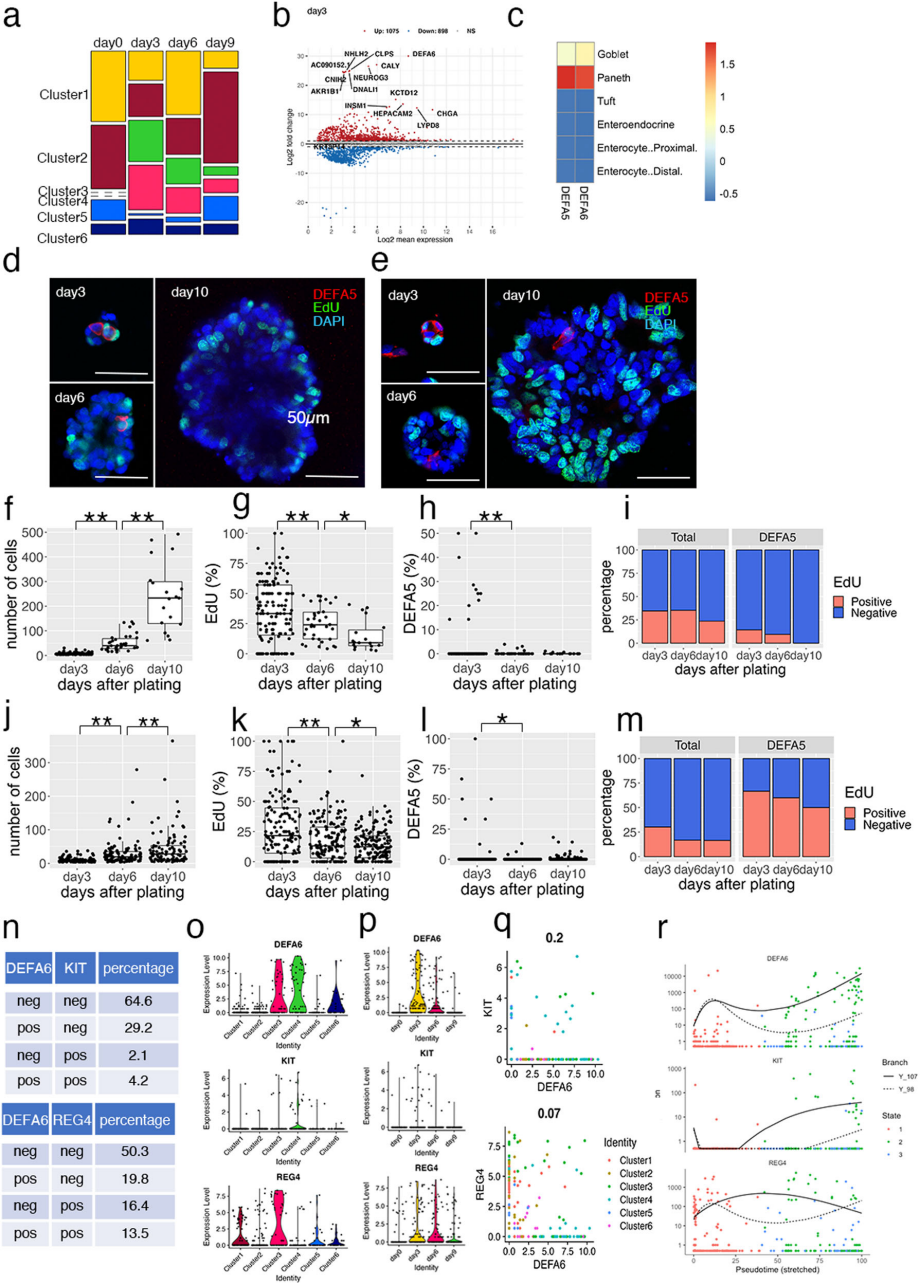
Induction of Paneth-like cells during organoid reconstitution. **a** Enrichment plot showing the altered proportions of clusters during organoid culture. Cell-type clusters were determined using uniform manifold approximation and projection (UMAP) as shown in Fig. 2a; the proportions of each cluster are shown. **b** MA plot showing the top 15 most significant differentially expressed genes (DEGs) between days 0 and 3 after single cell plating. **c** Overlap of DEGs of defensin alpha 5 (DEFA5)- and DEFA6-expressing cells and gene markers of intestinal epithelial cells. The scale indicates the scaled −log10 (*p* value). **d**, **e** Representative images of immunofluorescent staining of HCT25-1 (**d**) and HCT45-1T (**e**). Organoids cultured for the indicated days were fixed and stained for DEFA5 (Red). Incorporated 5-ethynyl-2′-deoxyuridine (EdU) was visualized by the click reaction (green) and nuclei were stained using 4′,6-diamidino-2-phenylindole (DAPI; blue). Three-dimensional (3D) images were obtained; a representative single plane is shown. The bar denotes 50 µm. **f**–**m** Cell number (**f** and **j**), EdU^+^ cells (**g** and **k**), DEFA5^+^ cells per organoid (**h** and **l**), and percentage of EdU^+^ cells in DEFA5^+^ cells (**i** and **m**) of HCT26-1T (**f**–**i**) and HCT45-1T (**j**–**m**) were counted in the 3D images. In box-plots, center line and upper and lower quartiles indicated median and upper and lower quartiles, respectively. Whiskers indicated 1.5× interquartile range. Combined results of three replicates are shown; **p < 0.01 and *p < 0.05 (*t*-test). Comparative analysis of DEFA6^+^, KIT^+^, and REG4^+^ cells in colorectal cancer (CRC) patient-derived organoids (PDOs). Tables show the percentage of DEFA6 and KIT or DEFA6 and REG4 (**n**). Violin plots showing marker gene expression across clusters (**o**) and culture days (**p**). Scattered plot showing the correlation between DEFA6 and KIT (upper panel) and that between DEFA6 and REG4 (lower panel). Correlation coefficients are shown in (**q**). **r** Pseudotime analysis showing the expression levels during organoid reconstruction. Cells were colored according to the states shown in Fig. 2f.

Subsequently, we focused on the alterations in cellular composition during organoid reconstitution from single OLFM4^+^ cells (Fig. 3a). Enrichment plots showed that cells in clusters 3 and 4 were rarely detected on day 0, but comprised 62.5% of the cells on day 3. DEGs between days 0 and 3 contained well established secretory lineage cell marker genes, including CHGA, INSM1, and NEUROG3 (Fig. 3b). These observations indicate that the cell fate determination program shifted toward the secretory lineage after single-cell dissociation.

Notably, the canonical Paneth cell marker DEFA6 was identified as the most significant DEG (log2FC = 30, log2 mean expression=8.7), and DEFA5 was also upregulated (log2FC = 4.8, log2 mean expression=3.9). Paneth cells are located in the small intestine and are not normally found in the colon^20^. Thus, we characterized DEFA5- and DEFA6-expressing cells by calculating the overlaps between their DEGs and the marker genes of small intestinal epithelium cells determined by scRNA-seq of murine tissue^21^. Both DEFA5- and DEFA6-expressing cells shared molecular signatures with intestinal Paneth cells (Fig. 3c).

To validate the results of scRNA-seq analysis, we performed immunofluorescence staining of HCT25-1T (Fig. 3d). Owing to the lack of proven antibodies against DEFA6, we stained developing organoids with an antibody against DEFA5, and found that the total cell number per organoid increased during the observation period, whereas the number of 5-ethynyl-2′-deoxyuridine (EdU)-positive cells decreased (Fig. 3f, g). The percentage of DEFA5^+^ cells was 1.9% on day 3, and then decreased to 0.34% and 0.17% on days 6 and 10, respectively (Fig. 3h). Similar results were obtained using HCT45-1T (Fig. 3e, j-l). These observations support the notion that Paneth-like cells were induced in response to single-cell dissociation of organoids, although organoids were established from the colon, which lacks Paneth cells. Notably, on day 3, 14.3% of HCT25-1T and 66.7% of HCT45-1T of DEFA5^+^ cells were EdU^+^, suggesting they were not terminally differentiated but retained proliferation activity (Fig. 3i, m).

In the mouse colon, a subset of secretory lineage cells play a role equivalent to that of Paneth cells in the small intestine. These include c-Kit^+^ colonic crypt base secretory cells^9^ and Reg4^+^ deep crypt secretory cells^10^. In the human CRC-derived organoids, 4.2% and 13.5% of cells co-expressed DEFA6 and KIT or REG4, respectively (Fig. 3n). KIT^+^ and REG4^+^ cells were partitioned into clusters 3 and 4 (Fig. 3o) and their numbers increased on day 3 after plating (Fig. 3p). However, their expression profiles were not significantly correlated with that of DEFA6 (correlation coefficient = 0.2 and 0.07, for KIT+ and REG4+ cells, respectively) (Fig. 3q), and pseudotime analysis indicated that these cells were produced by a distinct differentiation process (Figs. 2g and 3r). Based on these observations, we conclude that DEFA6^+^ cells mainly represent a cell state distinct from those previously reported as colonic crypt base secretory cells or colon deep crypt secretory cells, although some cells co-expressed KIT or REG4.

### Notch signaling controls cell fate determination in CRC organoids

**Fig. 4:**
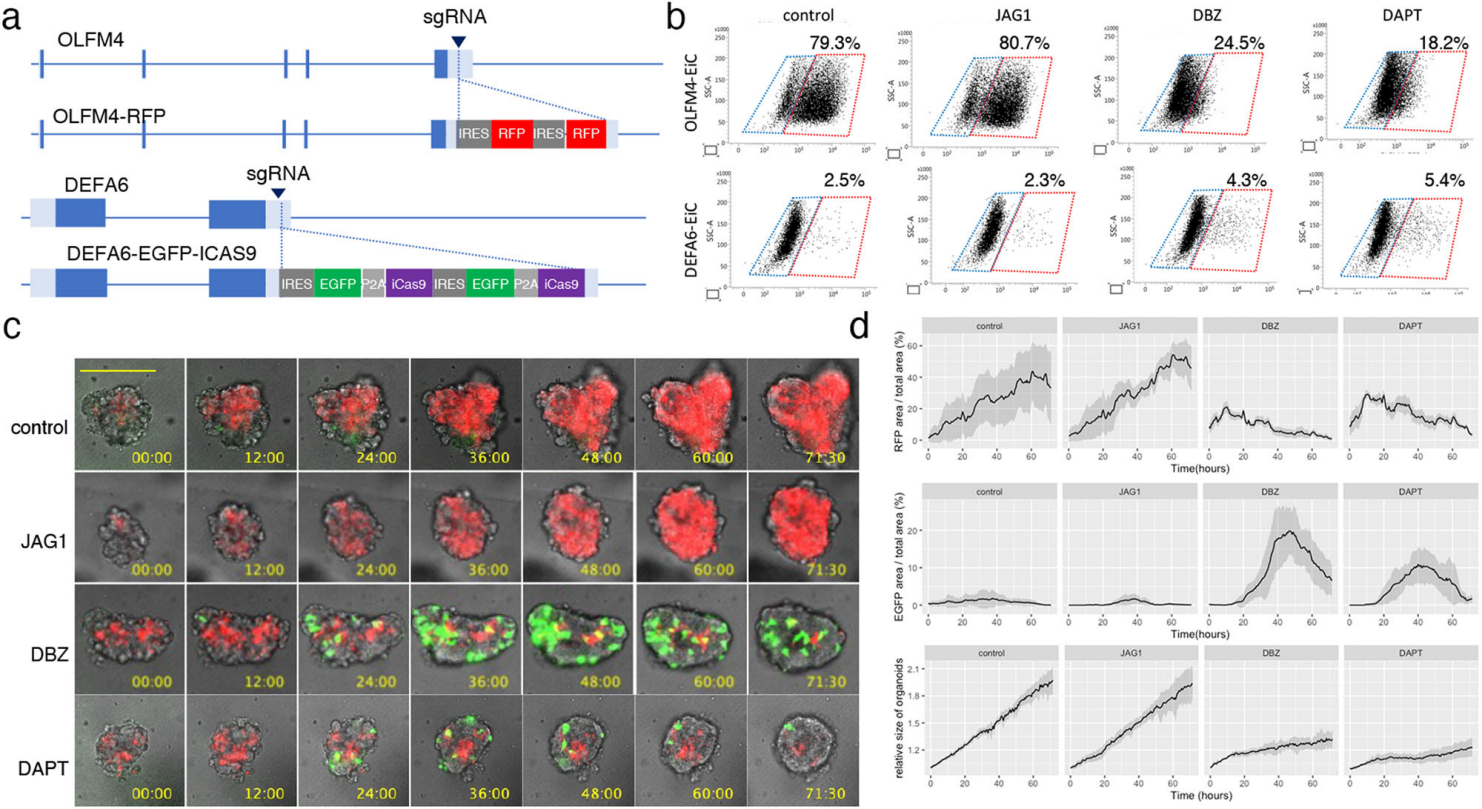
Visualization of OLFM4^+^ and DEFA6^+^ cells in patient-derived organoids (PDOs). **a** Internal ribosome entry site (IRES)-RFP and IRES-enhanced green fluorescence protein (EGFP)-P2A-inducible Caspase 9 (iCas9) cassettes were inserted into the 3’-untranslated region of olfactomedin 4 OLFM4 and defensin alpha 6 (DEFA6), respectively, generating PDOs harboring OLFM4-RFP/DEFA6-EGFP-iCas9. **b** Fluorescence-activated cell sorting (FACS) analysis showing the effects of Notch ligand and inhibitors. PDOs expressing OLFM4-EGFP-iCas9 (upper panels) and DEFA6-EGFP-iCas9 (lower panels) were cultured for 24 h followed by treatment with the indicated agents for 24 h. The percentage of EGFP^+^ cells was analyzed using fluorescence-activated cell sorting (FACS). **c** Time lapse images of PDOs. OLFM4-RFP/DEFA6-EGFP-iCas9 organoids were treated with the indicated agents, and videos were recorded every 30 min for 3 days. The bar denotes 100 µm. **d** Quantification of fluorescence images shown in (**c**). Data are shown as mean intensity ± standard deviation (*n* = 4).

Previously, we showed that OLFM4^+^ cells in PDOs harboring EiC in the OLFM4 locus could be reliably identified based on EGFP expression^12^. In this study, to analyze the temporal regulation of DEFA6^+^ cells developed from OLFM4^+^ cells, we generated PDOs in which the EiC and IRES-RFP (RFP) cassettes were integrated into the 3’ UTR of DEFA6 and OLFM4 genes, respectively (Fig. 4a and Supplementary Fig. 4a and b). FACS analysis revealed that two Notch inhibitors, dibenzazepine (DBZ) and DAPT, reduce EGFP^+^ cells in OLFM4-EiC by 79.3% to 24.5% and 18.2%, respectively, and increased DEFA6-EiC by 2.5% to 4.3% and 5.4%, respectively, although jagged 1, a Notch ligand, did not induce overt effects (Fig. 4b). Additionally, we inserted the RFP gene in the DEFA6 locus of PDOs carrying EiC in the OLFM4 locus, which was generated in a previous study^11^. A small number of DEFA6^+^ cells were detected in each organoid of both genetically modified PDOs (Supplementary Fig. 4c). BB homodimerizer treatment effectively removed OLFM4^+^ cells in OLFM4-EiC/DEFA6-RFP organoids, leading to organoid collapse. However, it did not induce overt effects on OLFM4-RFP/DEFA6-EiC organoids, demonstrating that DEFA6^+^ cells were dispensable for the maintenance of the established organoids.

Time-lapse imaging demonstrated that the number of OLFM4^+^ cells continuously increased during the first 3 days after replating, indicating that these cells were actively self-replicating in the organoid expansion phase (Fig. 4c, d). DBZ and DAPT reduced the number of OLFM4^+^ cells and increased the number of DEFA6^+^ cells. The growth of organoids was impaired and the expression of DEFA6 started to decrease 48 h after replating. These data indicate that DEFA6^+^ cells were induced by the perturbation of Notch signaling, providing evidence that the cell fate determination program of Paneth cells in the normal intestine is maintained in advanced CRC.

### Cell-cell interaction supports organoid formation

**Fig. 5:**
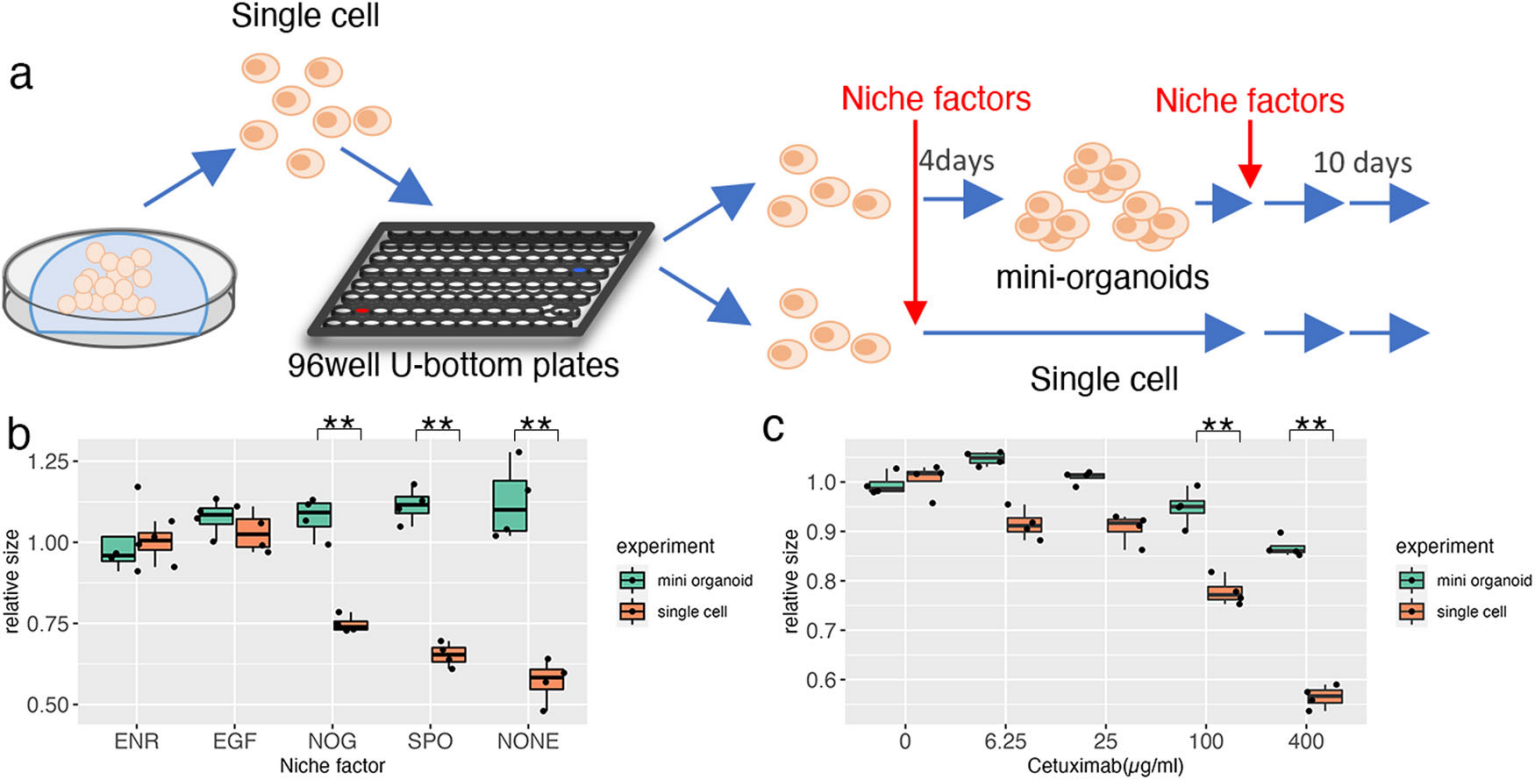
Niche factor dependency of patient-derived organoids (PDOs). **a** Schematic representation of the experiment. Single cells of PDOs were cultured in media containing test niche factors (single cell) or in ENR medium for 4 days to generate small clusters of cells followed by in-test media (mini-organoids). **b** Comparative analysis of niche factor dependency between single cells and mini-organoids. Organoids were cultured in media supplemented with the indicated niche factors for 10 days. The size of organoids was determined by analyzing at least 800 organoids and the relative size to that of organoids cultured in ENR medium is shown; **p < 0.01 (unpaired *t* test, *n* = 4 independent experiments). **c** Response to cetuximab. Organoids were cultured in media containing the indicated concentration of cetuximab and the size was determined as described in (**b**); **p < 0.01 (unpaired *t* test, *n* = 4 independent experiments).

In the normal intestine, Paneth cells provide niche factors that support Lgr5^+^stem cells^7^. Thus, we investigated whether the interaction between DEFA6^+^ cells contributed to the growth of OLFM4^+^ cells because the role of cellular interactions in advanced CRC has not been well established. The efficiency of organoid formation was analyzed under two different conditions: in mini-organoids and single cells (Fig. 5a). Mini-organoids, which consisted of an average of 6.8 cells (Fig. 3d, f), were generated by culturing single cells for 3 days. Mini-organoids grew in niche factor-free basal medium, whereas organoid formation from single cells was considerably impaired (Fig. 5b). These observations support the notion that cell-cell interactions in organoids derived from advanced CRC support organoid formation by providing niche factors.

Notably, epidermal growth factor (EGF) supported the growth of single cells. This suggests that cell–cell interactions in tumor tissues affect the efficacy of EGFR-targeting chemotherapeutic agents, which are commonly used for CRC treatment. This was tested using the clinically used drug cetuximab (Fig. 5c), which induced more substantial effects on single cells than on mini-organoids. HCT25-1T cells have a KRAS G13D mutation^11^. This mutation results in incomplete impairment of NF1 function, and the cells respond to EGFR inhibition^22,23^. In a clinical setting, cetuximab is effective against CRC carrying the KRAS G13D mutation^24^. These observations shed light on cell-cell interactions as potential therapeutic targets for improving the efficacy of cetuximab.

### DEFA6^+^ cells increase organoid formation efficiency

**Fig. 6:**
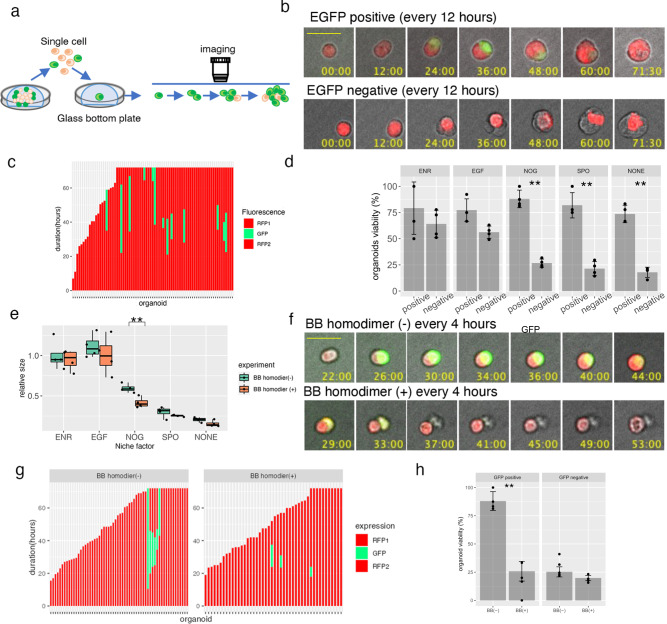
Timelapse imaging of organoid reconstruction. **a** Schematic representation of the experiment. OLFM4-RFP/DEFA6-EGFP-iCas9 organoids were dissociated into single cells and their growth was video recorded every 30 min for 3 days. **b** Representative video-recording of organoid reconstruction. Composite images of fluorescence and bright fields are shown. The organoid shown in the upper panel generated EGFP^+^ cells, while no EGFP^+^ cells were observed in the organoid shown in the lower panel. The bar denotes 25 µm. **c** Stacked bar plot showing the emergence of EGFP^+^ cells of OLFM4-RFP/DEFA6-EGFP-iCas9 organoids. Each bar represents one organoid generated from single RFP^+^ cells. Red and green indicate organoids containing RFP^+^ cells and RFP^+^/EGFP^+^ cells, respectively. **d** The percentage of alive organoids 3 days after plating of single RFP^+^ cells. At least 50 cells were counted in each condition. Data are shown as the mean ± standard deviation; **p < 0.01(unpaired *t*-test, *n* = 4 independent experiments). **e** Effect of BB homodimer on organoid growth in media containing different niche factors. Organoids were cultured in the presence (green) or absence (red) of BB homodimer in the indicated media for 10 days. The organoid size was determined by analyzing at least 800 organoids and the relative size to that of organoids cultured in ENR medium is shown; **p < 0.01 (unpaired *t*-test, *n* = 4 independent experiments). **f** Timelapse images of the organoids reconstituted from single OLFM4^+^ cells. Composite images of fluorescence and bright field are shown. The rapid elimination of EGFP+ cells by BB homodimer is evident. The base denotes 25 µm. **g** Stacked bar plot showing the generation of EGFP^+^ cells in OLFM4-RFP/DEFA6-EGFP-iCas9 organoids. Each bar represents one organoid generated from single RFP^+^ cells. Red and green indicate organoids containing RFP^+^ cells and RFP^+^/EGFP^+^ cells, respectively. **h** Percentage of live organoids 3 days after plating of single RFP^+^ cells. At least 50 cells were counted in each condition. Data are shown as the mean ± standard deviation; ** *p* < 0.01(unpaired *t*-test, *n* = 4 independent experiments).

Considering that DEFA6 is a canonical marker for Paneth cells that supports crypt basal stem cells in the normal intestine, we investigated whether DEFA6^+^ cells in advanced CRC had equivalent functions equivalent to those of Paneth cells. To compare the construction efficiency of DEFA6^+^ organoids to that of DEFA6^-^ organoids, HCT25-1T-OLFM4_RFP-DEFA6_EiC cells were dissociated into single cells, and organoid formation was monitored for 3 days (Fig. 6a). During this period, 17% of RFP^+^ cells produced EGFP^+^ cells in ENR medium (Fig. 6b, c). RFP^+^ cells also produced RFP^-^/EGFP^-^ cells, indicating that OLFM4^+^ cells produced progenies distinct from DEFA6^+^ cells (Fig. 6b, lower panels).

We investigated the efficiency of organoid formation under different media conditions (Fig. 6d). PDOs were maintained in media supplemented with EGF, R-spondin-1, and noggin, which support organoid growth of by controlling RAS, Wnt, and bone morphogenetic protein signaling, respectively. On average, 76.6% of DEFA6^+^ organoids were viable, irrespective of niche factors. The viability of DEFA6^-^ organoids was slightly reduced in the presence of ENR or EGF (60.1% and 56.2%, respectively), whereas it was dramatically reduced in medium supplemented with noggin, R-spondin-1, or in niche factor-free medium (28.7%, 21.8%, and 17.9%, respectively), consistent with the low efficiency of organoid formation of single cells in EGF-free media (Fig. 5b). These observations support the hypothesis that DEFA6^+^ cells support organoid formation.

### Ablation of DEFA6^+^ cells reduces organoid formation efficiency

To directly evaluate the role of DEFA6^+^ cells, we selectively ablated these cells during organoid formation. HCT25-1T-OLFM4_RFP-DEFA6-EiC cells were dissociated into single cells and cultured in the presence or absence of BB homodimerizer. Consistent with the reconstruction experiment, no significant effects were observed in ENR or EGF medium (Fig. 6e). Notably, significant reduction was evident in noggin-containing medium, but not in medium supplemented with R-spondin-1 or niche factor-free medium. These findings indicate that DEFA6^+^ cells provide a niche environment that is not replaced by noggin.

The ablation of DEFA6^+^ cells in noggin-containing medium was analyzed using time-lapse imaging. BB homodimerizer treatment quickly eliminated EGFP^+^ cells, demonstrating the efficient ablation of DEFA6^+^ cells (Fig. 6f). Furthermore, the treatment reduced the duration of EGFP expression (25.9 h and 11.7 h in the presence and absence of BB homodimerizer, respectively) and the percentage of EGFP^+^ cells (11.5% and 7.6% in the presence and absence of BB homodimerizer, respectively) (Fig. 6g). Notably, ablation of DEFA6^+^ cells induced death in OLFM4^+^ cells (Supplementary Fig. 5) and the viability of the DEFA6^+^ organoids was significantly reduced (Fig. 6h). These observations indicate that DEFA6^+^ cells support OLFM4^+^ cells and contribute to organoid formation, which recapitulates the role of Paneth cells in providing the niche environment of stem cells in the normal intestine.

### Identification of DEFA5/6^+^ cells in CRC surgical specimen

**Fig. 7:**
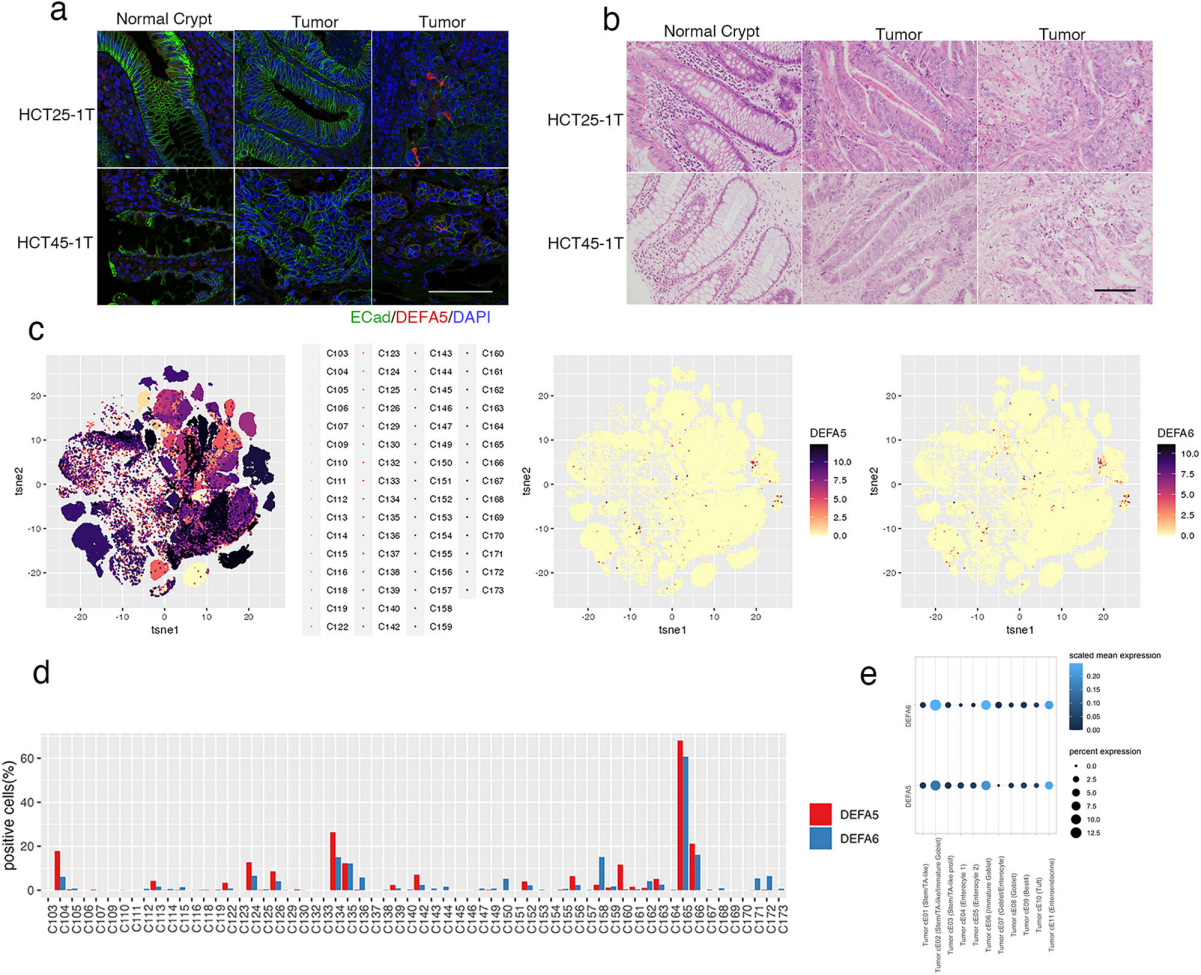
Paneth-like cells in tumor specimens. **a** Tissue slices of FFPE sample stained for defensin alpha 5 (DEFA5; red) and E-Cadherin (ECad; green). Nuclei are visualized using 4′,6-diamidino-2-phenylindole (DAPI; blue). ECad signals form normal crypts (left panels), tumor regions with strong (middle panels), and weak (right panels) are shown. The bar denotes 100 µm. **b** Hematoxylin and eosin staining of tissue slices. Normal crypt and tumor regions with (middle panels) or without (right panels) tubular structures are shown. The bar denotes 100 µm. **c** t-Distributed stochastic neighbor embedding of tumor cells by specimen (left panel), DEFA5^+^ cells (middle panel), and DEFA6^+^ cells (left panel). **d** Percentage of DEFA5^+^ (red) and DEFA6^+^ (blue) cells in each patient. **e** Dot plot showing scaled mean expression (color) and percent expression (dot size) of DEFA5 and six in each cluster.

The localization of Paneth-like cells in CRC tissue was histologically analyzed in the original surgical specimen from which the PDOs were established. Tumor sections were stained with antibodies to DEFA5 and epithelial cell adhesion molecule (ECad) to evaluate the integrity of tumor tissue (Fig. 7a). DEFA5^+^ cells were not detected in the normal crypt or in tumor tissues where cell adhesion was maintained, but were identified in areas where the ECad signal was reduced. Hematoxylin and eosin staining of this area revealed the loss of epithelial tissue integrity, although cells morphologically resembling Paneth cells were not found (Fig. 7b). Considering that Paneth-like cells were produced by single cell dissociation of organoids, these observations provide in vivo evidence supporting their induction in response to loss of cell-cell contact.

To evaluate the prevalence of Paneth-like cells in patients with CRC, we explored a previously reported scRNA-seq dataset^25^ (Fig. 7c). Out of 62 patients, DEFA5^+^ and DEFA6^+^ cells were detected in 41 (66.1%) and 51 (82.3%) cases, respectively (Fig. 7d). In 108,497 tumor cells that were analyzed, the overall percentage of positive cells was 3.6% and 3.1%, respectively. Gene expression programs identified by consensus non-negative matrix factorization revealed that their expression levels were high in Tumor cE02 (Stem/TA-like/Immature Goblet), Tumor cE06 (Immature Goblet) and cE11 (Enteroendocrine), demonstrating the association of Paneth-like cells with secretory lineage cells (Fig. 7e).

To further characterize the cells, we analyzed the 594 colorectal adenocarcinoma (COADREAD) patients listed in TCGA PanCancer Atlas^26^, and found that the expression levels of DEFA5 and 6 were correlated (*R*^2^ = 0.64) (Supplementary Fig. 6a). Comparative analysis between DEFA5/6 positive and negative patients (z-score > 2) revealed that their expression levels were not correlated to the specific tumor type (adenocarcinoma and mucinous) or location (colon, rectum) (qval = 0.038 and 0.038, respectively) (Supplementary Fig. 6b). Additionally, no significant difference was observed in mutation frequencies between DEFA5/6 positive and negative groups (qval = 0.906 and = 0.721, respectively) (Supplementary Fig. 6c). These observations indicated that a substantial fraction of Paneth-like cells in CRC expresses DEFA5 and 6, and that their induction is not histologically or genetically specified.

## Discussion

The normal intestine is maintained by a balance between self-renewal and lineage commitment of Lgr5^+^ stem cells, which is controlled by multiple pathways^5^ whose activities are modulated by somatic mutations that occur during tumorigenesis. However, their effects on cell fate determination in advanced CRC have not been fully elucidated. Using PDOs established from a patient with advanced CRC, we previously identified OLFM4 as the gene most strongly associated with the stem cell cluster^11^. In this study, we analyzed the cellular lineage produced from single OLFM4^+^ cells. Trajectory inference analysis revealed three cellular states with bifurcating lineages, demonstrating that cancer stem cells produced tumor cells resembling secretory and absorptive cells. Notably, the secretory lineage cells were preferentially produced after single-cell dissociation. DEFA6, a canonical marker of Paneth cells in normal intestine, was identified as the most prominently induced gene. These cells were histologically identified in surgical specimens and database analyses indicated their prevalence in CRC. As Paneth cells are thought to be localized in the small intestine, but not in normal colon, these findings may indicate the distinct cellular plasticity of tumor cells, which may contribute to maintaining the homeostasis of cancer tissue.

Among several markers of Paneth cells, DEFA6 expression does not require bacterial induction^27^. This mode of expression makes DEFA6 a useful marker for detecting Paneth-like cells in germ-free organoid cultures. In the normal intestine, Paneth cells are considered terminally differentiated cells, and their lifespan is estimated to be approximately 30 days^28^. We found that the GFP signal representing DEFA6 expression in PDOs derived from advanced CRC lasted for an average of 20 h, and that cells lost the signal without apparent signs of cell death (Fig. 6b). These observations suggest that the transcription of DEFA6 is not continuous and that it is activated periodically. Alternatively, cells may lose their expression of DEFA6 because they proceed to further differentiation or dedifferentiation. Furthermore, we found that Paneth-like cells in advanced CRC were associated with cluster 3, which represents cycling cells. A subset of DEFA5^+^ cells incorporated EdU 3 days after single cell dissociation (Fig. 3i and m). These observations highlight the distinct nature of Paneth-like cells in advanced tumors compared to Paneth cells in the normal intestine.

The PDOs used in this study were derived from adenocarcinoma developed in the sigmoid colon, where Paneth cells usually do not exist. Therefore, the induction of Paneth-like cells in advanced CRC is of particular interest. We found that these cells were rarely detected in established organoids but appeared in the early phases of organoid reconstruction from single OLFM4^+^ cells, raising the possibility that Paneth-like cells can be generated by cell fate conversion in response to alteration in tumor integrity. Considering that the upregulation of the Paneth cell signature was reported in cetuximab-treated PDXs^29^, further understanding of the regulation of Paneth-like cells may provide clues to controlling CRC relapse.

This study sheds light on the significance of cell-cell interaction between OLFM4^+^ cancer stem cells and Paneth-like cells on tumor homeostasis. However, human CRC is a heterogenic disease, and OLFM4^+^ cells do not function as cancer stem cells in a subset of CRC-derived organoids^11^. Furthermore, advanced CRC has distinct inter-tumor heterogeneity in somatic mutation, genetic background, and the origin of tumor cells, which control the efficacy of chemotherapeutic agents. Further comprehensive analysis of multiple PDOs and evaluation of their relevance to the original tumors is necessary to develop effective treatments and precisely predict treatment efficacy.

## Methods

### Organoid culture

PDOs were established from surgical specimens that were obtained from consenting patients^11^. All procedures were approved by the Research Ethics Board at the JFCR Cancer Institute (Tokyo, Japan). All ethical regulations relevant to human research participants were followed. Organoids were suspended in Matrigel (BD Biosciences), and 25 µl of this suspension was dispensed into 48-well plates. After Matrigel polymerization, organoids were overlaid with Advanced Dulbecco’s Modified Eagle Medium/F12 (Gibco) supplemented with 10 ng/ml EGF (Invitrogen), 10% noggin-conditioned medium, and 1 µg/ml R-spondin-1 (R&D systems) at 37 °C in an atmosphere of 5% O_2._ The medium was changed every 3 or 4 days. For FACS analysis (Fig. 1) and scRNA-seq (Figs. 2, 3), PDOs were cultured in media supplemented with EGF, noggin, and R-spondin-1. The media were changed 3 days before single-cell dissociation and collection.

### Genome editing

Genome editing of PDOs was performed by introducing donor vectors and the gRNA-Cas9 complexes^12^. The gRNA-expressing constructs were prepared by cloning a pair of annealed oligonucleotides into the BbsI site of pX330 (#422320; Addgene). The donor construct for the insertion of IRES-RFP was prepared by cloning the 20-bp sgRNA recognition sequence oligonucleotide into the AgeI/NotI and PacI/NheI sites of pCMMP-MCS-IRES-mRFP (#36972; Addgene). A donor construct for insertion of the IRES-EGFP-iCas9 cassette was prepared using pCMMP-MCS-IRES-EGFP-P2A-iCas9 (#178533; Addgene). HCT25-1T-OLFM4-EGFP-iCas9/DEFA6-RFP was generated by inserting the IRES-RFP cassette into the 3′-UTR of the DEFA6 gene of HCT25-1T-OLFM4-EGFP-iCas9^11^. HCT25-1T-OLFM4-IRES-RFP/DEFA6-EGFP-iCas9 cells were generated by inserting the IRES-EGFP-iCas9 cassette into the 3′UTR of DEFA6, followed by insertion of the IRES-RFP cassette into the 3′ UTR of OLFM4.

The donor construct, sgRNA, and Cas9 expression vector were electrophoresed using the PiggyBac system^30^, and transfected organoids were selected using 2 µg/ml puromycin or 800 µg/ml G418 5 days after electroporation. Genomic DNA was obtained using lysis buffer (10 mM Tris-HCl, pH 8.0, 25 mM EDTA, 1% SDS, 150 mM NaCl, 100 µg/ml proteinase K), followed by phenol-chloroform extraction and ethanol precipitation. Southern blot analysis was performed using standard procedures and a digoxigenin (DIG) system (Roche).

### FACS analysis

PDOs were dissociated using TrypLE Express (Life Technology), and undigested cell clusters were removed using a 20-µm cell strainer (Falcon). The cells were washed twice with phosphate-buffered saline (PBS) supplemented with 0.2% bovine serum albumin (BSA) and 2 mM EDTA, and stained with 7-aminoactinomycin D (BD Biosciences). The cells were sorted using a 100-µm nozzle (Aria III, BD Bioscience), and 1 × 10^4^ cells were embedded in 25 µl of Matrigel and cultured in a 48-well plate. Gating strategy for flow cytometric analysis in Fig. 1 is shown in Supplementary Fig. 7.

### scRNA-seq

PDOs were dissociated using TrypLE Express, and undigested cell clusters were removed using a 20-µm cell strainer. Live cells were collected using FACS and their concentration was adjusted to 300 cells/µl in PBS containing 0.2% BSA and 2 mM EDTA. Sorted cells were mixed with C1 suspension reagent (Fluidigm) and loaded on a 10–17 µm C1 cell integrated fluidic circuit chip (IFC, Fluidigm). The captured cells were inspected by microscopy (BZ-X710, KEYENCE). cDNA amplification was performed using the SMARTer Ultra Low RNA kit for the Fluidigm C1^TM^ System (Clontech) on the C1 Single-Cell Auto Prep IFC according to the manufacturer’s instructions. The amplified cDNA was quantified using a 2100 Bioanalyzer (Agilent Technologies), and a library was constructed using the Nextera XT DNA Sample Preparation kit (Illumina). The sequence was outsourced to Cancer Precision Medicine (Kanagawa, Japan), and each single-cell cDNA was sequenced at 150 pair-ends in the Novaseq SP-XP setting.

Fastq files were generated using FastQC, which were aligned using HISAT2 against human GRCh38 as a reference. Differential expression analysis on the gene-by-cell count matrix of time series scRNA-seq data was performed using the R package Seurat (ver3)^31^. Preprocessing of the count matrix data was performed using the SCTransform function. Pseudotime analysis and trajectory inference were performed using the monocle package (ver2.0)^32^. DEGs of DEFA5^+^ and DEFA6^+^ cells were obtained using the FindMakers function, and their overlap with intestinal markers was calculated using GeneOverlap packages (https://bioconductor.org/packages/release/bioc/html/GeneOverlap.html).

scRNA-seq expression data for Human Colon Atlas (c295) were downloaded from Single Cell Portal (https://singlecell.broadinstitute.org/single_cell). We used marix.barcodes.tsv, matrix.genes.tsv and matrix.mtx.gz for downstream analysis.

### TCGA analysis

We analyzed and visualized Colorectal Adenocarcinoma data (TCGA, PanCancer Atlas) using cBioPortal^33,34^.

### Immunofluorescence staining and imaging

PDOs were fixed in 4% paraformaldehyde in PBS for 30 min at room temperature and then permeabilized with 0.5% Triton X-100 for 1 h and blocked with 3% donkey serum in PBS for 1 h. PDOs were incubated overnight at 4 °C with an anti-DEFA5 antibody (A18208; ABclonal), followed by a Cy3-conjugated anti-rabbit antibody (AP132C; Sigma-Aldrich). Proliferating cells were detected by incorporation of EdU using a Click-iT EdU proliferation kit (Thermo Fisher Scientific). Cell nuclei were stained with 20 μg/ml 4′,6-diamidino-2-phenylindole (Invitrogen) in PBS for 15 min, and three-dimensional imaging was performed using a CellVoyager 8000 (Yokogawa) equipped with a 40× water immersion objective. Z-planes spanning a range of 100 µm were acquired using 4 µm z-steps. Images were analyzed using CellPathfinder (Yokogawa).

Formalin-fixed paraffin-embedded sections were deparaffinized with xylene followed by hydration with graded concentrations of ethanol. After antigen retrieval, samples were incubated with anti-DEFA5 antibody and anti-E-Cadherin antibody (610182, BD Bioscience), followed by a Cy3-conjugated anti-rabbit antibody and a FITC-conjugated anti-mouse antibody (55493, Cappel).

### Cell viability analysis

PDOs were dissociated using TrypLE Express, and undigested cell clusters were removed using a 20-µm cell strainer. Cells were collected by centrifugation at 1000 × *g* for 5 min, washed with basal medium, and counted. A total of 2000 cells were added to 10 µl of Matrigel and dispensed into a 96 U-bottom plate (163320; Thermo Fisher Scientific, 163320). To generate mini-organoids, the cells were incubated for 4 days. To analyze niche factor dependency, cells were incubated in a test medium supplemented with niche factor combinations. Cetuximab sensitivity was tested in medium containing EGF, noggin, and R-spondin-1, and the medium was changed every 3 or 4 days. Cell viability was evaluated based on the organoid size determined using Cell3imager (Screen), which correlated well with the results of the ATP-based viability assay^35^.

### Timelapse imaging

To analyze the established organoids, 20 organoids in 10 µl Matrigel were dispensed into a four-well glass bottom 35 mm dish (D14100; Iwaki), and images were recorded using the CellVoyager CV 1000 system (Yokogawa) equipped with a 10× dry objective lens. Images were analyzed using the CellPathfinder software.

To analyze organoid reconstitution, single cells were prepared using TrypLE as described above, and 2000 cells in 10 µl Matrigel were dispensed into a four-well glass bottom 35 mm dish. Timelapse imaging was performed using the CellVoyager CV1000 equipped with a 20× water immersion objective. Z planes spanning a range of 20 µm were acquired using 10-µm z-steps. Composites of maximum intensity projection of fluorescence images and bright-field images were generated using the package implemented in CellVoyger 1000, and images were analyzed using Image J (ver2.3.0).

### Statistics and reproducibility

Statics and reproducibility. The results are expressed as mean ± standard deviation (SD). Statistical analysis when comparing two groups was performed using Studint’s *t* test.

### Reporting summary

Further information on research design is available in the Nature Portfolio Reporting Summary linked to this article.

### Supplementary information

Supplementary Figures

Description of Additional Supplementary Files

Supplementary Data

Reporting Summary

## Data Availability

The scRNA-seq data of the organoid reconstitution experiment are available from DDBJ DRA database under accession number DRA017245. The row scRNA-seq data and the cell type annotations for the colorectal cancer data set^25^ are available in the GEO database under accession number GSE178341. The mutation RNA-seq data from the CRC samples^26^ are available at TCGA cancer atlas (https://gdc.cancer.gov/about-data/publications/pancanatlas). Any additional information required to reanalyze the data reported in this paper is available from the lead contact upon request. The numerical source data behind the graphs in the figures can be found in the Supplementary Data file.

## Code availability

The bioinformatics software used is R version 4.3.0. The packages used in our model and plotting code are as follows: ggplot2 3.3.5, Matrix1.5.4.1, GeneOverlap1.26.0. Seurat4.3.0.1.

## References

[1] Lytle NK, Barber AG, Reya T (2018). Stem cell fate in cancer growth, progression and therapy resistance. Nat. Rev. Cancer.

[2] Reya T, Morrison SJ, Clarke MF, Weissman IL (2001). Stem cells, cancer, and cancer stem cells. Nature.

[3] Vermeulen L, Sprick MR, Kemper K, Stassi G, Medema JP (2008). Cancer stem cells-old concepts, new insights. Cell Death Differ..

[4] Sancho R, Cremona CA, Behrens A (2015). Stem cell and progenitor fate in the mammalian intestine: Notch and lateral inhibition in homeostasis and disease. EMBO Rep..

[5] Beumer J, Clevers H (2021). Cell fate specification and differentiation in the adult mammalian intestine. Nat. Rev. Mol. Cell Biol..

[6] Fre S (2005). Notch signals control the fate of immature progenitor cells in the intestine. Nature.

[7] Sato T (2011). Paneth cells constitute the niche for Lgr5 stem cells in intestinal crypts. Nature.

[8] Serra D (2019). Self-organization and symmetry breaking in intestinal organoid development. Nature.

[9] Rothenberg ME (2012). Identification of a cKit(+) colonic crypt base secretory cell that supports Lgr5(+) stem cells in mice. Gastroenterology.

[10] Sasaki N (2016). Reg4+ deep crypt secretory cells function as epithelial niche for Lgr5+ stem cells in colon. Proc. Natl Acad. Sci. USA.

[11] Okamoto T (2021). Comparative Analysis of Patient-Matched PDOs Revealed a Reduction in OLFM4-Associated Clusters in Metastatic Lesions in Colorectal Cancer. Stem Cell Rep..

[12] Okamoto T, Natsume Y, Yamanaka H, Fukuda M, Yao R (2021). A protocol for efficient CRISPR-Cas9-mediated knock-in in colorectal cancer patient-derived organoids. STAR Protoc..

[13] Straathof KC (2005). An inducible caspase 9 safety switch for T-cell therapy. Blood.

[14] Gerbe F, Legraverend C, Jay P (2012). The intestinal epithelium tuft cells: specification and function. Cell Mol. Life Sci..

[15] Parikh K (2019). Colonic epithelial cell diversity in health and inflammatory bowel disease. Nature.

[16] Blache P (2004). SOX9 is an intestine crypt transcription factor, is regulated by the Wnt pathway, and represses the CDX2 and MUC2 genes. J. Cell Biol..

[17] Gierl MS, Karoulias N, Wende H, Strehle M, Birchmeier C (2006). The zinc-finger factor Insm1 (IA-1) is essential for the development of pancreatic beta cells and intestinal endocrine cells. Genes Dev..

[18] Schonhoff SE, Giel-Moloney M, Leiter AB (2004). Minireview: development and differentiation of gut endocrine cells. Endocrinology.

[19] Herring CA (2018). Unsupervised trajectory analysis of single-cell RNA-Seq and imaging data reveals alternative tuft cell origins in the gut. Cell Syst..

[20] Wallaeys C, Garcia-Gonzalez N, Libert C (2023). Paneth cells as the cornerstones of intestinal and organismal health: a primer. EMBO Mol. Med..

[21] Haber AL (2017). A single-cell survey of the small intestinal epithelium. Nature.

[22] McFall T (2019). A systems mechanism for KRAS mutant allele-specific responses to targeted therapy. Sci. Signal.

[23] Rabara D (2019). KRAS G13D sensitivity to neurofibromin-mediated GTP hydrolysis. Proc. Natl Acad. Sci. USA.

[24] De Roock W (2010). Association of KRAS p.G13D mutation with outcome in patients with chemotherapy-refractory metastatic colorectal cancer treated with cetuximab. JAMA.

[25] Pelka K (2021). Spatially organized multicellular immune hubs in human colorectal cancer. Cell.

[26] Hoadley KA (2018). Cell-of-origin patterns dominate the molecular classification of 10,000 tumors from 33 types of cancer. Cell.

[27] Putsep K (2000). Germ-free and colonized mice generate the same products from enteric prodefensins. J. Biol. Chem..

[28] Clevers HC, Bevins CL (2013). Paneth cells: maestros of the small intestinal crypts. Annu Rev. Physiol..

[29] Lupo B (2020). Colorectal cancer residual disease at maximal response to EGFR blockade displays a druggable Paneth cell-like phenotype. Sci. Transl. Med..

[30] Fujii M, Matano M, Nanki K, Sato T (2015). Efficient genetic engineering of human intestinal organoids using electroporation. Nat. Protoc..

[31] Butler A, Hoffman P, Smibert P, Papalexi E, Satija R (2018). Integrating single-cell transcriptomic data across different conditions, technologies, and species. Nat. Biotechnol..

[32] Qiu X (2017). Reversed graph embedding resolves complex single-cell trajectories. Nat. Methods.

[33] Cerami E (2012). The cBio cancer genomics portal: an open platform for exploring multidimensional cancer genomics data. Cancer Discov..

[34] Gao J (2013). Integrative analysis of complex cancer genomics and clinical profiles using the cBioPortal. Sci. Signal.

[35] Okamoto T (2022). Integration of human inspection and artificial intelligence-based morphological typing of patient-derived organoids reveals interpatient heterogeneity of colorectal cancer. Cancer Sci..

