# Supplementary material for: Paneth-like cells produced from OLFM4+ stem cells support OLFM4+ stem cell growth in advanced colorectal cancer
Source: Commun Biol. 2024 Jan 5;7:27. doi: 10.1038/s42003-023-05504-8 (PMC10770338; doi:10.1038/s42003-023-05504-8)
Supplement: Supplementary file 1 — Supplementary Figures [file 42003_2023_5504_MOESM1_ESM.pdf]

Supplementary Information

Paneth-like cells produced from OLFM4$^+$ stem cells support OLFM4$^+$ stem cell growth in advanced colorectal cancer

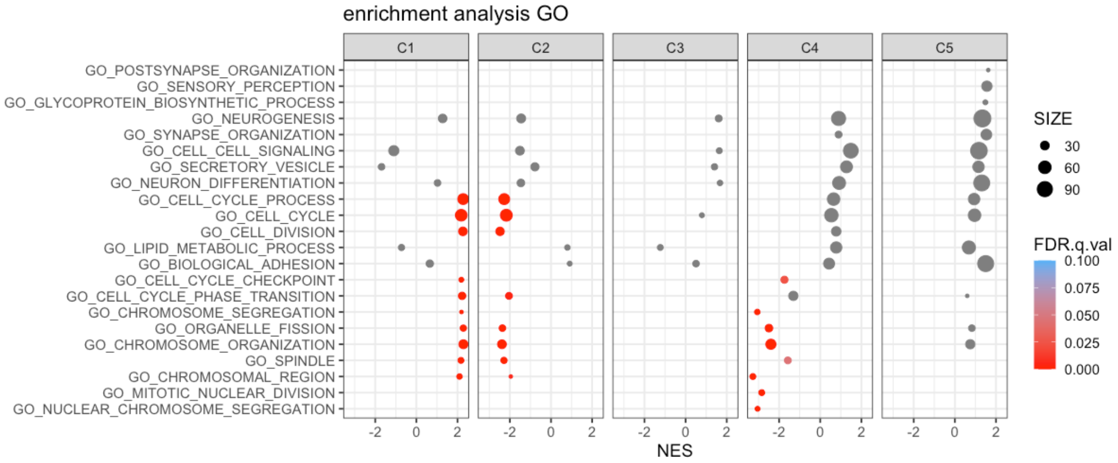

Supplementary Fig. 1 Dot plot of GSEA results. Significantly activated and suppressed pathways derived from the Gene Ontology gene subset in MsigDB in indicated clusters are listed. The color of the dots represents the FDR value, and the diameter represents the enriched gene count.

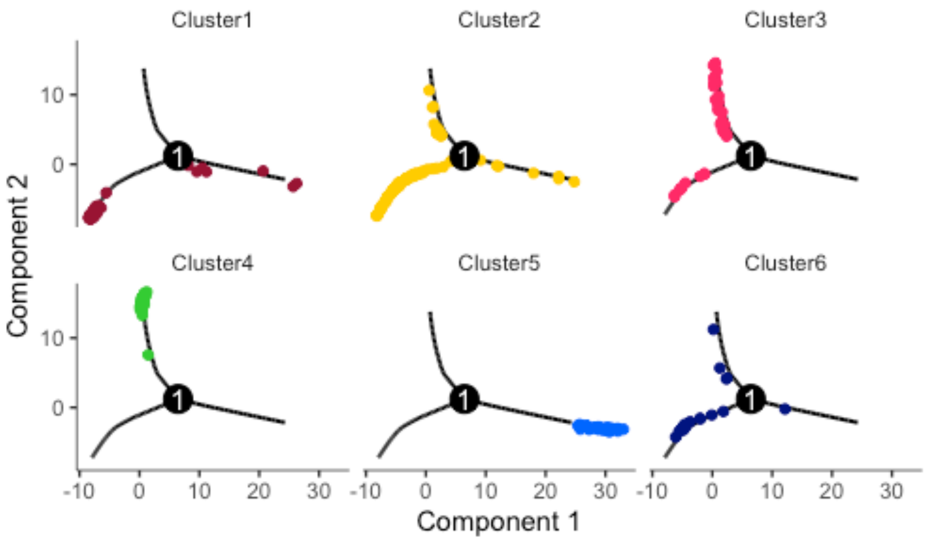

Supplementary Fig. 2 Trajectory analysis of advanced CRC patient-derived organoids (PDOs). Two-dimensional representation of the scRNA-seq data obtained at day 0, three, six and nine after single cell dissociation. Cells are colored according to the clusters shown in Figure 1A.

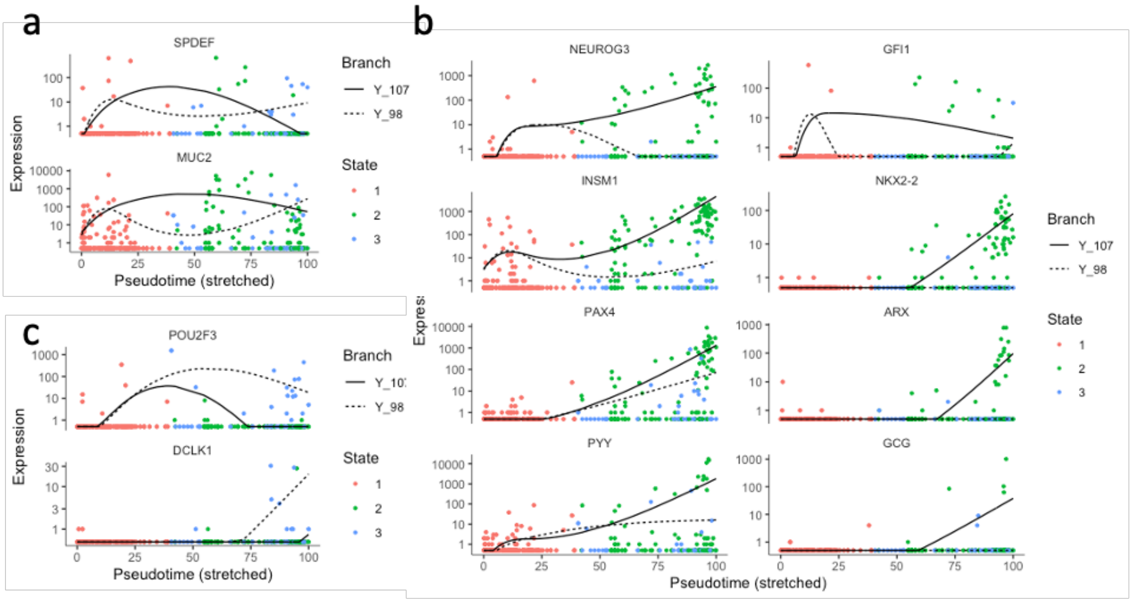

Supplementary Fig. 3 Pseudotime analysis showing the expression levels during organoid reconstruction Pseudotime kinetics of indicated genes from the root to two outcomes were shown. Cells were colored according to states shown in Figure 2F. Canonical markers for goblet cells (a), endocrine cells (b) and tuft cells (c) were shown.

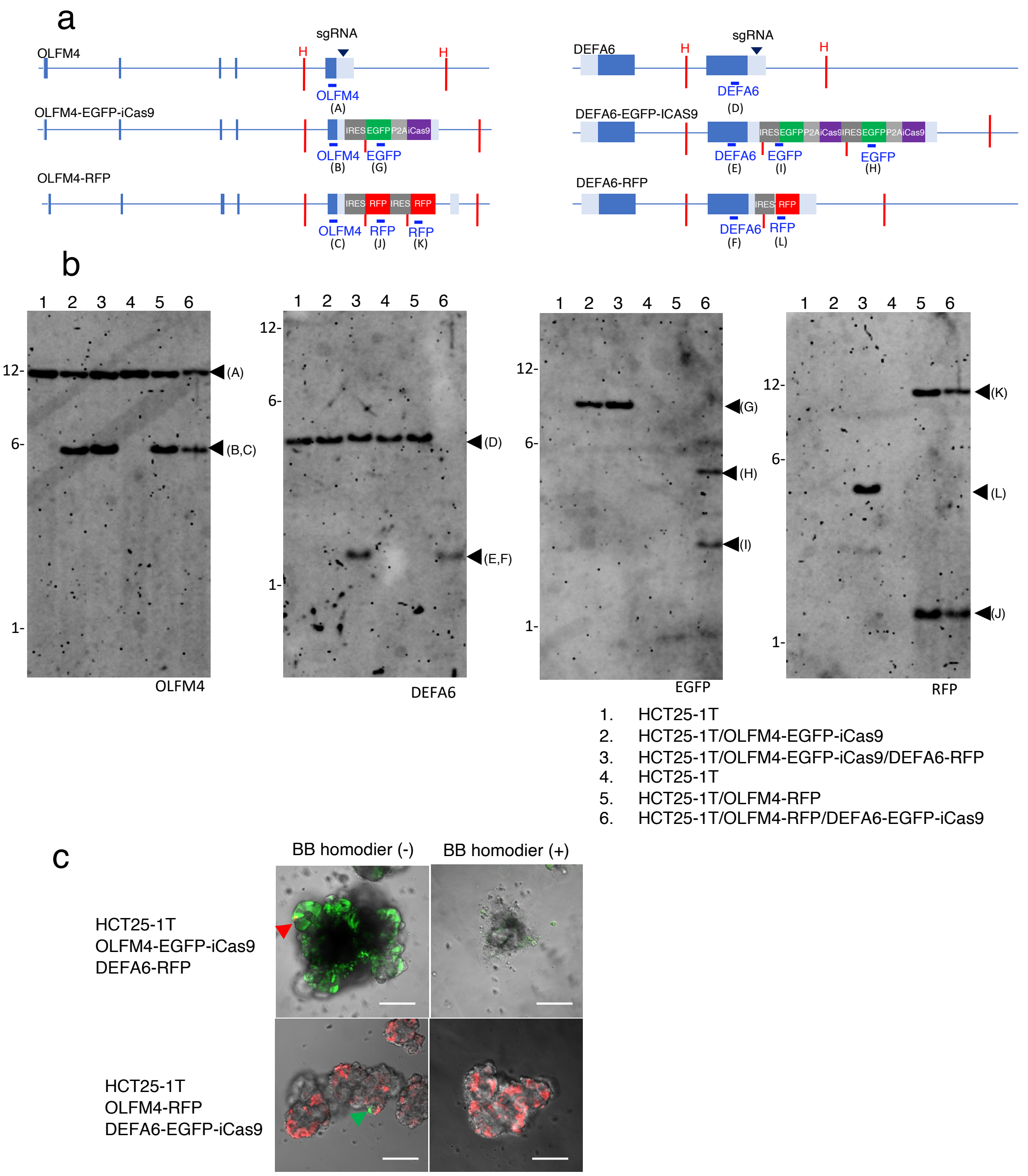

Supplementary Fig. 4 Genome editing of PDOs. (a) The IRES-EGFP-P2A-iCas9 and IRES-RFP cassettes were inserted into the 3' UTR of OLFM4 or DEFA6. HindIII sites and the probes used for Southern blot analysis and the expected signal sizes are shown in red, blue and black respectively. (b) Southern blot analysis of genome-edited PDOs. Genome DNA were isolated from indicate PDOs, digested with HindIII, and analyzed using probes indicated in (a). The probes and signal sizes are shown. (c) Ablation of OLFM4^{+} cells (upper panels) or DEFA6^{+} cells (lower panels). HCT25-1T/OLFM4-

EGFP-iCas9/DEFA6-RFP or HCT25-1T/OLFM4-RFP/DEFA6- EGFP-iCas9 organoids were treated with 2.5 nM BB homodier (AP20187) for 3 day. Bar=200 μm.

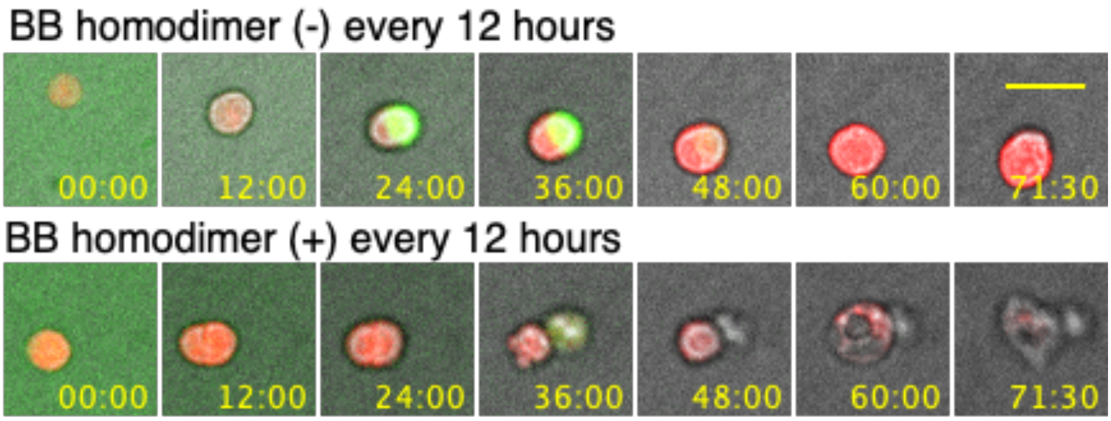

Supplementary Fig. 5 Timelapse imaging of organoid reconstruction.
Representative video-recording of the organoid reconstruction. HCT25-1T/OLFM4-RFP/DEFA6- EGFP-iCas9 organoids were digested and single cells were cultured in Matrigel in the absence (upper panels) or presence (lower panels) of BB homodimer for three days. Composite images of fluorescence and bright field are shown. Cells in upper panels express EGFP for 12-24 hours, whereas those in lower panels quickly underwent cell death, and the organoid eventually collapsed. Bar=25 µm.

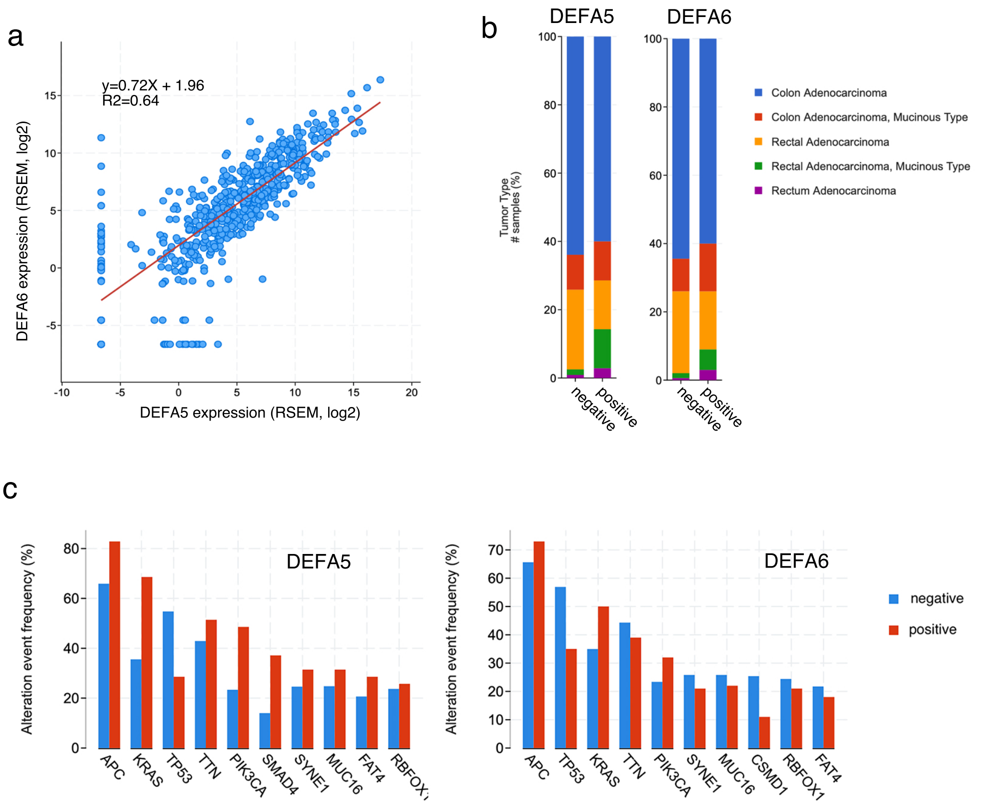

Supplementary Fig. 6 Identification of DEFA5^{+} and 6^{+} cells in TCGA dataset. (a) Correlated expression between DEFA5 and 6. (b) Tumor type specificity of DEFA5 and 6 expressing cells. specimen were classified into negative and positive by their expression (z-score >2), and visualized their tumor types. (c) comparison of mutation profile between positive and negative in DEFA5 and DEFA6 expression. Specimen was classified as described in (b) and the frequency of most frequently mutated genes were shown.

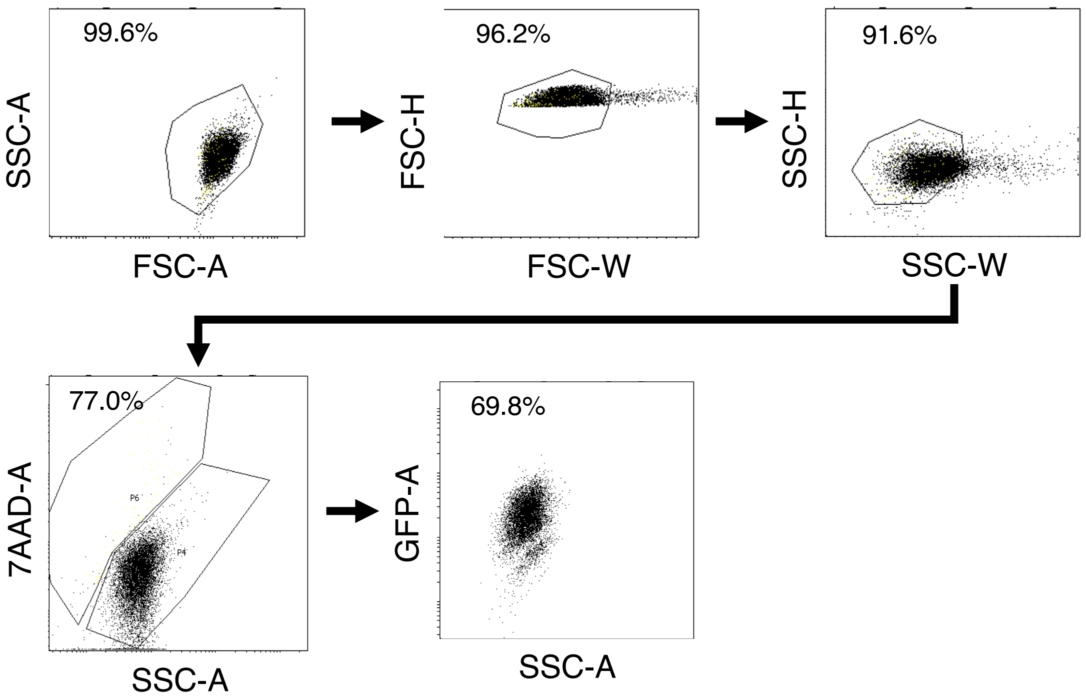

Supplementary Fig. 7 Gating strategies Gating strategies for flow cytometric analysis of patient derived organoids in Figure 1.
