# Supplementary material for: Paneth-like cells produced from OLFM4+ stem cells support OLFM4+ stem cell growth in advanced colorectal cancer
Source: Commun Biol. 2024 Jan 5;7:27. doi: 10.1038/s42003-023-05504-8 (PMC10770338; doi:10.1038/s42003-023-05504-8)
Supplement: Supplementary file 2 — Description of Additional Supplementary Files [file 42003_2023_5504_MOESM2_ESM.pdf]

Description of Additional Supplementary Files

File name: Supplementary Data
Description: Numerical source data behind the graphs in the manuscript.
