# Supplementary material for: Paneth-like cells produced from OLFM4+ stem cells support OLFM4+ stem cell growth in advanced colorectal cancer
Source: Commun Biol. 2024 Jan 5;7:27. doi: 10.1038/s42003-023-05504-8 (PMC10770338; doi:10.1038/s42003-023-05504-8)
Supplement: Supplementary file 4 — Reporting Summary [file 42003_2023_5504_MOESM4_ESM.pdf]

nature portfolio

Corresponding author(s): DAPR
Last updated by author(s): 2023/10/19

Reporting Summary

Nature Portfolio wishes to improve the reproducibility of the work that we publish. This form provides structure for consistency and transparency in reporting. For further information on Nature Portfolio policies, see our Editorial Policies and the Editorial Policy Checklist.

Statistics

For all statistical analyses, confirm that the following items are present in the figure legend, table legend, main text, or Methods section.

n/a	Confirmed	
☐	☒	The exact sample size (*n*) for each experimental group/condition, given as a discrete number and unit of measurement
☐	☒	A statement on whether measurements were taken from distinct samples or whether the same sample was measured repeatedly
☐	☒	The statistical test(s) used AND whether they are one- or two-sided<br>*Only common tests should be described solely by name; describe more complex techniques in the Methods section.*
☒	☐	A description of all covariates tested
☒	☐	A description of any assumptions or corrections, such as tests of normality and adjustment for multiple comparisons
☐	☒	A full description of the statistical parameters including central tendency (e.g. means) or other basic estimates (e.g. regression coefficient) AND variation (e.g. standard deviation) or associated estimates of uncertainty (e.g. confidence intervals)
☐	☒	For null hypothesis testing, the test statistic (e.g. *F*, *t*, *r*) with confidence intervals, effect sizes, degrees of freedom and *P* value noted<br>*Give P values as exact values whenever suitable.*
☒	☐	For Bayesian analysis, information on the choice of priors and Markov chain Monte Carlo settings
☒	☐	For hierarchical and complex designs, identification of the appropriate level for tests and full reporting of outcomes
☒	☐	Estimates of effect sizes (e.g. Cohen's *d*, Pearson's *r*), indicating how they were calculated

Our web collection on statistics for biologists contains articles on many of the points above.

Software and code

Policy information about availability of computer code

Data collection	No software was used for data collection.
Data analysis	The bioinformatics software used are R version 4.3.0. The packages used in our model and plotting code are as follows: ggplot2 3.3.5, Matrix1.5.4.1, GeneOverlap1.26.0. Seurat4.3.0.1.

For manuscripts utilizing custom algorithms or software that are central to the research but not yet described in published literature, software must be made available to editors and reviewers. We strongly encourage code deposition in a community repository (e.g. GitHub). See the Nature Portfolio guidelines for submitting code & software for further information.

Data

Policy information about availability of data

All manuscripts must include a data availability statement. This statement should provide the following information, where applicable:

- Accession codes, unique identifiers, or web links for publicly available datasets
- A description of any restrictions on data availability
- For clinical datasets or third party data, please ensure that the statement adheres to our policy

The scRNA-seq data of the organoid reconstitution experiment is available from DDBJ DRA data base under accession number DRA017245. The row scRNA-seq data and the cell type annotations for the colorectal cancer data set 25 are available in the GEO database under accession number GSE178341. The mutation RNA-seq data from the CRC samples 26 are available at TCGA cancer atlas (https://gdc.cancer.gov/about-data/publications/pancanatlas).

Any additional information required to reanalyze the data reported in this paper is available from the lead contact upon request.
The scRNA-seq data of the organoid reconstitution experiment is available from DDBJ DRA data base under accession number DRA017245. The row scRNA-seq data and the cell type annotations for the colorectal cancer data set 25 are available in the GEO database under accession number GSE178341. The mutation RNA-seq data from the CRC samples 26 are available at TCGA cancer atlas (https://gdc.cancer.gov/about-data/publications/pancanatlas).
Any additional information required to reanalyze the data reported in this paper is available from the lead contact upon request.

Research involving human participants, their data, or biological material

Policy information about studies with human participants or human data. See also policy information about sex, gender (identity/presentation), and sexual orientation and race, ethnicity and racism.

Reporting on sex and gender	This is not a sex- or gender-based analysis.
Reporting on race, ethnicity, or other socially relevant groupings	Any confounding variables on race, ethnicity, or other socially relevant grouping were no used in this study.
Population characteristics	Covariate-relevant population characteristics are not relevant in this study.
Recruitment	Self-selection bias does not occur, because genome-edited PDOs established in previous study were used.
Ethics oversight	JFCR Cancer Insitute

Note that full information on the approval of the study protocol must also be provided in the manuscript.

Field-specific reporting

Please select the one below that is the best fit for your research. If you are not sure, read the appropriate sections before making your selection.

☒ Life sciences ☐ Behavioural & social sciences ☐ Ecological, evolutionary & environmental sciences

For a reference copy of the document with all sections, see nature.com/documents/nr-reporting-summary-flat.pdf

Life sciences study design

All studies must disclose on these points even when the disclosure is negative.

Sample size	This study analyzed two samples which were established in previous study.
Data exclusions	No data were excluded.
Replication	All attempts at replication were successful.
Randomization	This is not relevant to this study, because samples were not allocated into experimental groups.
Blinding	This is not relevant to this study, because samples were not allocated into experimental groups.

Reporting for specific materials, systems and methods

We require information from authors about some types of materials, experimental systems and methods used in many studies. Here, indicate whether each material, system or method listed is relevant to your study. If you are not sure if a list item applies to your research, read the appropriate section before selecting a response.

Materials & experimental systems

n/a	Involved in the study	
☐	☒	Antibodies
☒	☐	Eukaryotic cell lines
☒	☐	Palaeontology and archaeology
☒	☐	Animals and other organisms
☒	☐	Clinical data
☒	☐	Dual use research of concern
☒	☐	Plants

Methods

n/a	Involved in the study	
☒	☐	ChIP-seq
☐	☒	Flow cytometry
☒	☐	MRI-based neuroimaging

Antibodies

Antibodies used	anti DEFA5 (A18208, ABclonal)
Validation	https://abclonal.co.jp/index.php?s=/catalog-antibodies/DEFA5RabbitpAb/A18208

Plants

Seed stocks	*Report on the source of all seed stocks or other plant material used. If applicable, state the seed stock centre and catalogue number. If plant specimens were collected from the field, describe the collection location, date and sampling procedures.*
Novel plant genotypes	*Describe the methods by which all novel plant genotypes were produced. This includes those generated by transgenic approaches, gene editing, chemical/radiation-based mutagenesis and hybridization. For transgenic lines, describe the transformation method, the number of independent lines analyzed and the generation upon which experiments were performed. For gene-edited lines, describe the editor used, the endogenous sequence targeted for editing, the targeting guide RNA sequence (if applicable) and how the editor was applied.*
Authentication	*Describe any authentication procedures for each seed stock used or novel genotype generated. Describe any experiments used to assess the effect of a mutation and, where applicable, how potential secondary effects (e.g. second site T-DNA insertions, mosiacism, off-target gene editing) were examined.*

Flow Cytometry

Plots

Confirm that:

☒ The axis labels state the marker and fluorochrome used (e.g. CD4-FITC).

☒ The axis scales are clearly visible. Include numbers along axes only for bottom left plot of group (a 'group' is an analysis of identical markers).

☒ All plots are contour plots with outliers or pseudocolor plots.

☒ A numerical value for number of cells or percentage (with statistics) is provided.

Methodology

Sample preparation	Enzymatically dissociated single cells of PDOs harboring the IRES-EGFP-P2A-iCas9 cassette in the 3’ UTR of the OLFM4 gene.
Instrument	BD FACS Aria III
Software	BD FACSDiva
Cell population abundance	The population of EGFP-negative cells were first determined by sorting the parental PDOs, and that of EGFP-positive cells were calculated by analyzing those that exhibited higher EGFP signal.
Gating strategy	Single cells were obtained by sequential primary gating of FSC-A/SSC-A, FSC-W/FSC-H, and SSC-W/SSC-H. Dead cells were eliminated using 7AAD staining, followed by GFP expression. The boundary was defined by using parental PDOs that do not carry EGFP gene.

☒ Tick this box to confirm that a figure exemplifying the gating strategy is provided in the Supplementary Information.
